# The effects of conjugated linoleic acid supplementation on lipid profile in adults: A systematic review and dose–response meta-analysis

**DOI:** 10.3389/fnut.2022.953012

**Published:** 2022-11-03

**Authors:** Omid Asbaghi, Damoon Ashtary-larky, Kaveh Naseri, Saeede Saadati, Mohammad Zamani, Mahnaz Rezaei Kelishadi, Maryam Nadery, Saeid Doaei, Neda Haghighat

**Affiliations:** ^1^Cancer Research Center, Shahid Beheshti University of Medical Sciences, Tehran, Iran; ^2^Student Research Committee, Shahid Beheshti University of Medical Sciences, Tehran, Iran; ^3^Nutrition and Metabolic Diseases Research Center, Ahvaz Jundishapur University of Medical Sciences, Ahvaz, Iran; ^4^Gastroenterology and Liver Diseases Research Center, Research Institute for Gastroenterology and Liver Diseases, Shahid Beheshti University of Medical Sciences, Tehran, Iran; ^5^Department of Medicine, School of Clinical Sciences, Monash University, Melbourne, VIC, Australia; ^6^Department of Clinical Nutrition, School of Nutritional sciences and Dietetics, Tehran University of Medical Sciences, Tehran, Iran; ^7^Department of Community Nutrition, School of Nutrition and Food Science, Isfahan University of Medical Sciences, Isfahan, Iran; ^8^Department of Dietetics and Nutrition, Robert Stempel College of Public Health & Social Work, Florida International University, Miami, FL, United States; ^9^Reproductive Health Research Center, Department of Obstetrics and Gynecology, Al-Zahra Hospital, School of Medicine, Guilan University of Medical Sciences, Rasht, Iran; ^10^Laparoscopy Research Center, Shiraz University of Medical Sciences, Shiraz, Iran

**Keywords:** conjugated linoleic acid, lipid profile, systematic review, meta-analysis, CLA

## Abstract

**Background:**

The findings of trials investigating the effect of conjugated linoleic acid (CLA) administration on lipid profile are controversial. This meta-analysis of randomized controlled trials (RCTs) was performed to explore the effects of CLA supplementation on lipid profile.

**Methods:**

Two authors independently searched electronic databases including PubMed, Web of Science, and Scopus until March 2022, in order to find relevant RCTs. The random effects model was used to evaluate the mean and standard deviation.

**Results:**

In total, 56 RCTs with 73 effect sizes met the inclusion criteria and were eligible for the meta-analysis. CLA supplementation significantly alter triglycerides (TG) (WMD: 1.76; 95% CI: −1.65, 5.19), total cholesterols (TC) (WMD: 0.86; 95% CI: −0.42, 2.26), low-density lipoprotein cholesterols (LDL-C) (WMD: 0.49; 95% CI: −0.75, 2.74), apolipoprotein A (WMD: −3.15; 95% CI: −16.12, 9.81), and apolipoprotein B (WMD: −0.73; 95% CI: −9.87, 8.41) concentrations. However, CLA supplementation significantly increased the density lipoprotein cholesterol (HDL-C) (WMD: −0.40; 95% CI: −0.72, −0.07) concentrations.

**Conclusion:**

CLA supplementation significantly improved HDL-C concentrations, however, increased concentrations of TG, TC, LDL-C, apolipoprotein A, and apolipoprotein B.

**Systematic review registration:**

https://www.crd.york.ac.uk/prospero/#recordDetails, identifier: CRD42022331100.

## Introduction

Dyslipidemia has been shown to be a major predictor of cardiovascular disease (CVD), atherosclerosis, and type 2 diabetes mellitus (T2DM) (1–3), characterized by either one or a combination of the elevated serum concentration of total cholesterol (TC), total triglyceride (TG), low-density lipoprotein cholesterol (LDL-C), and the reduced serum concentration of high-density lipoprotein cholesterol (HDL-C) (1, 4). An imbalance between LDL-C and HDL-C can lead to increasing the incidence rate of myocardial infarction (MI) and stroke (5). Additionally, as result of the accumulation of plaque within the arteries, atherosclerotic CVD can be raised when LDL-C level is higher than normal (5). However, HDL-C has a protective role against atherosclerotic CVD (6).

Hypercholesterolemia is the most prevalent form of dyslipidemia, being the 15th leading cause of death in 1990, growing to 11th in 2007 and 8th in 2019 (7). According to the latest reports, the prevalence of hypercholesterolemia among adults was as follows: in Europe (53.7%), America (47.7%), South East Asia (30.3%), and Africa (23.1%) (8). The worldwide burden of dyslipidemia has raised over the previous 30 years (7), accounting for more than 4 million deaths annually (4). Accordingly, CVD as one of the primary outcomes of dyslipidemia presents a tremendous economic burden on the healthcare system (9), and T2DM has also been demonstrated to raise health care costs (10).

The major risk factors related to the development of dyslipidemia are poor dietary habits and sedentary lifestyle, overweight and obesity (11, 12), and alcohol consumption and cigarette smoking (13, 14). Over the past few years, although dietary interventions have been carried out to control dyslipidemia, adherence to strict long-term dietary restrictions can be challenging. Thus, dietary supplements can be an efficient approach in addition to lifestyle interventions (15–19).

Conjugated linoleic acid (CLA) is a collective term that refers to a heterogeneous group of geometric isomers of linoleic acid; up to 28 isomer forms are detected, of them “c9,t11” and “t10,c12” are especially important (20). CLA is naturally found in the fat, milk, and meat of ruminant animals such as cow, sheep, and goat (20, 21). Accumulating studies have shown the effects of CLA on the management of lipid abnormalities related to CVD and T2DM (22). The underlying mechanisms of action of CLA are through lipid metabolism, modifying enzyme activity, and hormonal profile (23). CLA can increase lipolysis in adipocytes, diminish fatty acids synthesis, and reduce lipogenesis. Moreover, CLA can increase beta-oxidation of mitochondrial fatty acids and as a result decreases triacylglycerol synthesis (23–25).

Apolipoprotein A (Apo A) is considered as a major structural protein of high-density lipoprotein, and Apolipoprotein B (Apo B) is the primary protein constituted of low-density lipoprotein (26). Apo B is an independent risk predictor for the severity of coronary artery disease (CAD) (26). In a study on the effect of CLA supplementation on serum level of Apo A and Apo B, there were no changes in these factors in healthy female young individuals (27). A review and meta-analysis study, included 23 randomized controlled trial (RCT), showed that CLA supplements caused a significant reduction in LDL-C level (28). In line with this study, Santurino et al. demonstrated that the consumption of PUFA n-3 and CLA naturally enriched goat cheese on 68 overweight and obese subjects for 12 weeks significantly increase the HDL-C (29). However, in a double-blind, randomized, placebo-controlled trial on 401 overweight or obese participants investigating the effect of 6-month CLA supplementation (2.5 g/day c9, t11 CLA + 0.6 g/day c12 t10 CLA) on the clinical parameters related to atherosclerosis like plasma lipids, there were no significant effects of CLA on serum concentrations of TG, TC, LDL-C, and HDL-C (30). In another small trial, 2-month of 3 g/day mixed CLA supplementation on patients with coronary artery disease (CAD) had no effect on the plasma TG, LDL-C, or HDL-C (31). Also, Fouladi et al. showed that a 12-week CLA plus exercise intervention among overweight adults has no effects on serum concentration of TG and LDL-C (32). Thus, a large number of human trials have failed to demonstrate a protective effect of CLA against CVD risk factors focusing on lipid profile.

According to previous findings, the general impact of CLA on lipid profile is equivocal, thereby demonstrating the need for a comprehensive systematic review and meta-analysis of clinical trials on this topic. Therefore, given the unclear impact of CLA on plasma lipid concentration, the aim of the current meta-analysis was to investigate the effects of CLA on lipid profile in adults.

## Methods and materials

This study was carried out according to Preferred Reporting Items for Systematic Reviews and Meta-Analyses (PRISMA) protocol for reporting systematic reviews and meta-analysis (33). And also, the research question of the systematic review is clearly defined in terms of populations (adults), interventions (CLA), comparators (control-group), outcomes (lipid profile), and study designs (RCTs) (PICOS).

### Search strategy

We conducted a comprehensive literature search in the online databases of PubMed/MEDLINE, Scopus, Web of Science, and Cochrane library up to 22 March. We applied the following MeSH and non-MeSH terms in the search strategy:

(“Conjugated linoleic acid” OR “conjugated fatty acid” OR “bovic acid” OR “rumenic acid” OR “CLA”) AND (Intervention OR “Intervention Study” OR “Intervention Studies” OR “controlled trial” OR randomized OR randomized OR random OR randomly OR placebo OR “clinical trial” OR Trial OR “randomized controlled trial” OR “randomized clinical trial” OR RCT OR blinded OR “double blind” OR “double blinded” OR trial OR “clinical trial” OR trials OR “Pragmatic Clinical Trial” OR “Cross-Over Studies” OR “Cross-Over” OR “Cross-Over Study” OR parallel OR “parallel study” OR “parallel trial”).

There were no restrictions on time and language of publications. Additionally, to prevent missing any publications, all the references of the related papers were checked. All searched studies were included in the Endnote software for screening; consequently, duplicate citations and unpublished manuscripts were removed.

### Inclusion criteria and exclusion criteria

The inclusion criteria for the current study were: (1) randomized controlled clinical trials, (2) studies on adult population (age >18 y), (3) studies that administered CLA in different forms including “c9, t11” and “t10,c12” isomers supplement and food enriched CLA, (4) RCTs with at least 1 week's duration of trial, and (5) controlled trials that reported mean changes and their standard deviations (SDs) of lipid profile throughout the trial for both intervention and control groups or presented required information for calculation of those effect sizes. If there was more than 1 published article for one dataset, the more complete set was included. Clinical trials with an extra intervention group were considered as 2 separate studies. The exclusion criteria in the current meta-analysis were experimental studies, those with a cohort, cross-sectional, and case–control design, review articles, and ecological studies. Also, trials without a placebo or control group and those which were not randomized, and/or performed on children and adolescents, were excluded.

### Data extraction

Two independent reviewers (MR and DA) completed data extraction from each qualified RCTs. Extracted data contain the name of the first author; publication year; location of the study; study design; sample size in each group; individuals' characteristics such as mean age, sex, and BMI; the CLA dose used for intervention; duration of intervention; mean changes; and SDs of lipid profile markers throughout the trial for both intervention and control groups, and the confounding variables adjusted in the analysis. If data were reported in different units, we converted them to the most frequently used unit.

### Quality assessment

The quality of qualified studies was measured by two independent researchers (OA and DA) by using the Cochrane Collaboration modified risk of bias tool, in which the risk of bias in RCTs is assessed in seven domains, including random sequence generation, allocation concealment, reporting bias, performance bias, detection bias, attrition bias, and other sources of bias (34). As a result, terms as “Low,” “High,” or “Unclear” were used to evaluate each domain (Table 1).

**Table 1:** Risk of bias assessment.

**Studies**	**Random sequence generation**	**Allocation concealment**	**Selective reporting**	**Other sources of bias**	**Blinding (participants and personnel)**	**Blinding (outcome assessment)**	**Incomplete outcome data**	**General quality**
Blankson et al. (35)	L	L	H	H	L	U	L	Moderate
Berven et al. (36)	L	H	H	H	L	U	L	Low
Benito et al. (37)	L	H	L	L	H	H	L	Low
Mougios et al. (38)	L	H	H	L	L	U	L	Moderate
Riserus et al. (39)	L	H	H	H	L	U	L	Low
Noone et al. (40)	L	H	H	L	L	U	L	Moderate
Risérus et al. (41)	L	H	H	L	L	U	H	Low
Kamphuis et al. (42)	L	H	H	H	L	U	L	Low
Whigham et al. (43)	L	H	H	L	L	U	L	Moderate
Moloney et al. (44)	L	H	H	L	L	U	L	Moderate
Gaullier et al. (45)	U	H	H	H	L	U	L	Low
Riserus et al. (46)	L	H	H	H	L	U	H	Low
Song et al. (47)	L	H	H	H	L	U	L	Low
Desroches et al. (48)	L	H	H	L	H	H	L	Low
Gaullier et al. (45)	L	L	H	L	L	L	L	High
Tricon et al. (49)	L	H	H	L	L	U	L	Moderate
Naumann et al. (50)	L	H	H	L	L	U	L	Moderate
Colakoglu et al. (27)	L	H	L	H	H	H	H	Low
Schmitt et al. (51)	L	H	H	L	L	U	L	Moderate
Taylor et al. (52)	L	H	H	H	L	U	H	Low
Attar-Bashi etval (53)	L	L	H	L	L	U	L	High
Nazare et al. (54)	L	H	H	H	L	U	L	Low
Gaullier et al. (55)	L	L	H	H	L	U	L	Moderate
Steck et al. (56)	L	H	H	H	L	U	L	Low
Watras et al. (57)	L	L	H	H	L	U	H	Low
Lambert et al. (58)	L	H	H	H	L	U	L	Low
Iwata et al. (59)	L	H	H	L	L	U	L	Moderate
Park et al. (60)	L	H	H	H	L	U	L	Low
Aryaeian et al. (61)	L	H	H	H	L	U	L	Low
Raff et al. (62)	L	H	H	L	L	U	L	Moderate
Kim et al. (63)	L	H	H	L	L	U	L	Moderate
Son et al. (64)	L	L	H	L	L	U	L	High
Zhao et al. (65)	L	H	H	H	L	U	L	Low
Shadman et al. (66)	L	H	H	H	L	L	L	Low
Sofi et al. (67)	L	H	H	H	H	H	L	Low
Wanders et al. (68)	L	H	H	L	H	H	L	Low
Michishita et al. (69)	L	L	H	H	L	U	L	Moderate
Sluijs et al. (30)	L	L	H	L	L	U	L	High
Venkatramanan et al. (70)	L	H	H	L	H	H	L	Low
Brown et al. (71)	L	H	H	L	H	H	H	Low
Sato et al. (72)	L	H	H	L	L	U	L	Moderate
Joseph et al. (73)	L	H	H	H	L	U	H	Low
Pfeuffer et al. (74)	L	H	H	H	L	U	H	Low
Rubin et al. (75)	L	L	H	H	L	U	L	Moderate
Chen et al. (76)	L	L	H	L	L	U	L	High
Carvalho et al. (77)	L	H	H	H	l	U	L	Low
Lopez-Plaza et al. (78)	L	L	H	L	L	U	L	High
Bulut et al. (79)	L	H	L	H	L	U	L	Moderate
Shadman et al. (80)	L	H	H	L	L	U	L	Moderate
Jenkins et al. (81)	L	L	H	L	L	U	L	High
Eftekhari et al. (31)	L	H	H	L	H	H	L	Low
Baghi et al. (82)	L	H	H	L	L	U	L	High
Ebrahimi-Mameghani et al. (83)	L	L	H	L	L	L	L	High
Ribeiro et al. (84)	L	L	H	L	L	U	L	High
Fouladi et al. (32)	L	L	H	L	H	H	H	Low
Chang et al. (21)	L	L	H	H	L	U	L	Moderate

*General Low quality > 2 high risk, General moderate quality = 2 high risk, General high quality < 2 high risk.

### Statistical analysis

For obtaining the overall effect sizes, mean changes and their SDs of each variable in the CLA and control groups were applied. In case mean changes were not reported, we calculated them by considering changes in each outcome's values during the intervention. We also converted standard errors (SEs), 95% confidence intervals (CIs), and interquartile ranges (IQRs) to SDs using the method of Hozo et al. (85) to acquire the overall effect sizes, we used a random-effects model that takes between-study variations into account. Heterogeneity was determined by the *I*^2^ statistic and Cochrane's Q test. *I*^2^ value >50% or *p* <0.05 for the Q-test was characterized as significant between-study heterogeneity (86, 87). Subgroup analyses were carried out to find probable sources of heterogeneity based on the predefined variables including duration of intervention (≥12 *vs*. <12 weeks), intervention dose (≥3 *vs*. <3 g/day), participants' health condition (healthy and unhealthy), baseline serum levels of TG (≥150 *vs*. <150 mg/dl), TC (≥200 *vs*. <200 mg/dl), LDL-C (≥100 *vs*. <100 mg/dl), and HDL-C (≥50 *vs*. <50 mg/dl), and baseline levels of BMI (normal, overweight, and obese). To determine the non-linear effects of CLA dosage (g/day) on each variable concentration, fractional polynomial modeling was used. Sensitivity analysis was applied to detect the dependency of the overall effect size on a specific study. The possibility of publication bias was investigated by Egger's regression test and the formal test of Begg. The meta-analysis was conducted by the use of the STATA^®^ version 14.0 (StataCorp., College Station, Lakeway, TX, USA). *P-*value <*0.0*5 was considered as significant level.

### Certainty assessment

The overall certainty of evidence across the studies was graded based on the guidelines of the GRADE (Grading of Recommendations Assessment, Development, and Evaluation) Working Group. The quality of evidence was classified into four categories, according to the corresponding evaluation criteria: high, moderate, low, and very low (88).

## Results

### Study selection

**Figure 1:**
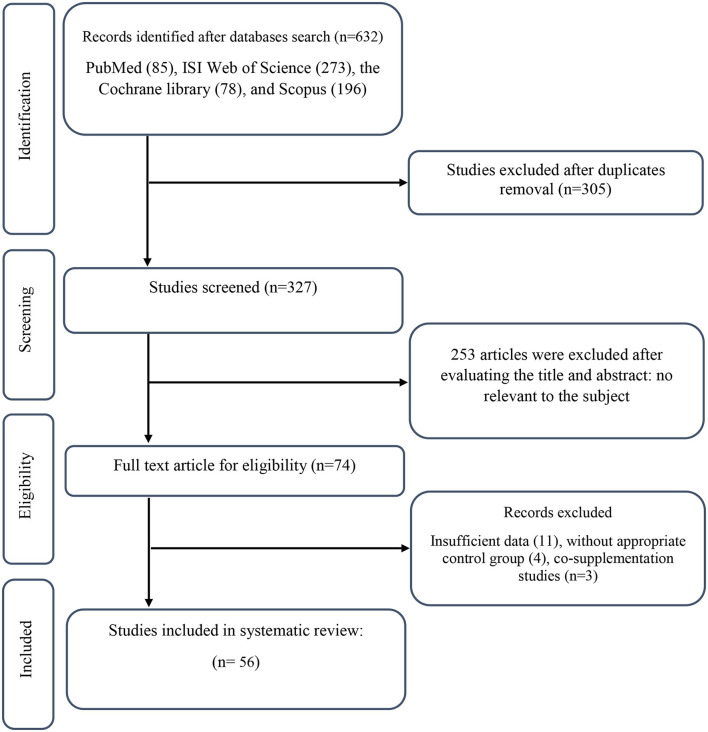
PRISMA flow diagram.

We found a total of 632 studies from our initial search in databases. After duplicate publications removal, 327 records remained, out of which 253 articles were identified as unrelated when screening based on title and abstract. Next, 74 suitable articles were candidate for full-text assessment. Out of these, 11 records due to not-reported lipid profile components, 4 records for not having control group, and 3 co-supplementation records were excluded. Finally, 56 eligible RCTs were included in our systematic review and meta-analysis. Figure 1 illustrates a summary of the study selection.

### Characteristics of the included studies

The characteristics of 56 RCTs included in the current systematic review and meta-analysis are shown in Table 2. In total, 73 effect sizes were extracted from 56 RCTs, including a total of 3,262 participants (1,773 participants in the CLA group and 1,738 ones in the placebo group). These RCTs were published between 2000 and 2020, from Asia (*n* = 18) (21, 27, 31, 32, 59–61, 63–66, 69, 72, 76, 79, 80, 82, 83), Europe (*n* = 26) (30, 35, 36, 38–42, 44–47, 49–52, 54–56, 62, 67, 68, 74, 75, 78, 89), America (*n* = 10) (37, 43, 48, 57, 70, 71, 73, 77, 81, 84), Africa (*n* = 1) (58), and Oceania (*n* = 1) (53). All RCTs enrolled both genders except 13 studies that were conducted exclusively on male (39, 41, 46, 48, 49, 59, 62, 73–75, 79, 81, 82) and 7 studies were performed on female (27, 37, 63, 64, 71, 77, 84). The mean age of individuals was between 18 and 68 years old with BMI range of 19–35.27 kg/m^2^. The dosage of CLA varied from 1.17 to 28.9 g/day and duration of intervention differed from 2 to 104 weeks across included RCTs. These studies were conducted in T2DM (44, 51, 66, 80), hypertension (65), metabolic syndrome (41, 77), hyperlipidemia (70, 73), rheumatoid arthritis (61), atherosclerosis (31), and others in healthy individuals. All studies employed a parallel design except 7 studies that applied cross-over design (48, 49, 67, 68, 70, 73, 75).

**Table 2:** Characteristics of the included studies.

**Studies**	**Country**	**Study design**	**Participant**	**Sex**	**Sample size**		**Trial duration (week)**	**Means age**		**Means BMI**		**Intervention**	
					**IG**	**CG**		**IG**	**CG**	**IG**	**CG**	**CLA dose**	**Control group**
Blankson et al. (35)	Norway	Paralell, R, PC, DB	Overweight and obese human	M/F (F: 15, M: 6)	11	10	12	44.3 ± 12.7	44.4 ± 13.2	30.3 ± 2.9	28 ± 2.4	6.8	Placebo
Blankson et al. (35)	Norway	Paralell, R, PC, DB	Overweight and obese human	M/F (F: 16, M: 6)	12	10	12	47.2 ± 13.5	44.4 ± 13.2	29.7 ± 2.5	28 ± 2.4	1.7	Placebo
Blankson et al. (35)	Norway	Paralell, R, PC, DB	Overweight and obese human	M/F (F: 13, M: 5)	8	10	12	42.8 ± 10.4	44.4 ± 13.2	27.7 ± 2.1	28 ± 2.4	3.4	Placebo
Blankson et al. (35)	Norway	Paralell, R, PC, DB	Overweight and obese human	M/F (F: 15, M: 6)	11	10	12	47.7 ± 11.3	44.4 ± 13.2	29.4 ± 2.8	28 ± 2.4	5.1	Placebo
Berven et al. (36)	Norway	Paralell, R, PC, DB	Obese human volunteers	M/F (F: 17, M: 30)	25	22	12	47.6 ± 7.1	46.5 ± 7	29.4 ± 2.6	30.1 ± 2.2	3.4	Placebo
Benito et al. (37)	USA	Paralell, R, PC, SB	Healthy	F: 17	7	10	8	27 ± 5.6	29.3 ± 6.8	23.6 ± 1.5	21.9 ± 31	3.9	Control diet
Mougios et al. (38)	Greece	Paralell, R, PC, DB	Healthy	M/F (F: 10, M: 14)	10	12	8	22.4 ± 1.7	22 ± 1.3	23.8 ± 2.7	22.7 ± 3.3	1.4	Placebo
Riserus et al. (39)	Sweden	Paralell, R, PC, DB	Obese middle-aged men	M: 24	14	10	4	54 ± 5.7	52 ± 7.8	32.2 ± 3.4	31.7 ± 1.9	4.2	Placebo
Noone et al. (40)	Ireland	Paralell, R, PC, DB	Healthy human subjects	M/F (F: 21, M: 13)	16	18	8	33.22 ± 11.78	32.31 ± 10.86	23.51 ± 3.1	23.35 ± 3.35	3	Control diet
Noone et al. (40)	Ireland	Paralell, R, PC, DB	Healthy human subjects	M/F (F: 18, M: 17)	17	18	8	28.58 ± 6.08	32.31 ± 10.86	24.08 ± 7.08	23.35 ± 3.35	3	Control diet
Risérus et al. (41)	Sweden	Paralell, R, PC, DB	Obese men with the metabolic syndrome	M: 38	19	19	12	51 ± 7.1	53 ± 10.1	30.1 ± 1.8	30.2 ± 1.8	3.4	Placebo
Risérus et al. (41)	Sweden	Paralell, R, PC, DB	Obese men with the metabolic syndrome	M: 38	19	19	12	55 ± 7.1	53 ± 10.1	31.2 ± 2.5	30.2 ± 1.8	3.4	Placebo
Kamphuis et al. (42)	Netherlands	Paralell, R, PC, DB	Overweight subjects	M/F (F: 14, M: 13)	13	14	13	36.2 ± 7.6	34 ± 9.1	26.2 ± 1.7	25.7 ± 1.4	3.6	Placebo
Kamphuis et al. (42)	Netherlands	Paralell, R, PC, DB	Overweight subjects	M/F (F: 14, M: 13)	14	13	13	40.9 ± 5	39.5 ± 7.7	25.6 ± 1.1	26.1 ± 1.4	1.8	Placebo
Whigham et al. (43)	USA	Paralell, R, PC, DB	Obese humans	M/F (F: 35, M: 15)	27	23	52	43.4 ± 4.8	41.2 ± 5.9	32 ± 2.1	31.4 ± 2.3	6	Placebo
Moloney et al. (44)	United Kingdom	Paralell, R, PC, DB	Type 2 diabetes mellitus	M/F: 32	16	16	8	63.8 ± 8.8	58.1 ± 10.8	29.1 ± 4	30.7 ± 4.8	3	Control diet
Gaullier et al. (45)	Norway	Paralell, R, PC, DB	Healthy overweight humans	M/F (F: 98, M: 21)	60	59	52	48 ± 10.7	45 ± 9.5	28.3 ± 1.6	27.7 ± 1.7	4.5	Placebo
Gaullier et al. (45)	Norway	Paralell, R, PC, DB	Healthy overweight humans	M/F (F: 98, M: 22)	61	59	52	44.5 ± 10.7	45 ± 9.5	28.1 ± 1.5	27.7 ± 1.7	4.5	Placebo
Riserus et al. (46)	Sweden	Paralell, R, PC, DB	Obese men	M: 25	13	12	12	54 ± 5.5	56 ± 6	30.6 ± 2	30.4 ± 2.5	3	Placebo
Gaullier et al. (45)	Norway	Paralell, R, PC, DB	Healthy overweight humans	M/F (F: 74, M: 14)	47	41	104	48.6 ± 10.6	45.1 ± 8.8	28.3 ± 1.5	27.4 ± 1.7	3.4	Placebo
Song et al. (47)	United Kingdom	Paralell, R, PC, DB	Young healthy volunteers	M/F (F: 20, M: 8)	14	14	12	31.8 ± 6.88	30.9 ± 7.14	24.3 ± 3.8	24.23 ± 3.69	3	Control diet
Desroches et al. (48)	Canada	Crossover, R, PC, B	Overweight and obese	M: 17	17	17	4	36.6 ± 12.4	36.6 ± 12.4	31.2 ± 4.4	31.2 ± 4.4	4.22	Control diet
Gaullier et al. (45)	Norway	Paralell, R, PC, DB	Healthy overweight humans	M/F (F: 69, M: 18)	46	41	104	45.1 ± 10.5	45.1 ± 8.8	28.1 ± 1.4	27.4 ± 1.7	3.4	Placebo
Tricon et al. (49)	United Kingdom	Crossover, R, PC, DB	Healthy middle-aged men	M: 32	32	32	6	45.5 ± 8.7	45.5 ± 8.7	25 ± 3.4	25 ± 3.4	1.4	Control diet
Naumann et al. (50)	Netherlands	Paralell, R, PC, DB	Overweight subjects with LDL phenotype B	M/F: 53	19	34	13	55 ± 7	51 ± 9	29.3 ± 2.4	28 ± 2.2	3	Control diet
Naumann et al. (50)	Netherlands	Paralell, R, PC, DB	Overweight subjects with LDL phenotype B	M/F: 68	34	34	13	51 ± 7	51 ± 9	28.6 ± 2.3	28 ± 2.2	3	Control diet
Colakoglu et al. (27)	Turkey	Paralell, R, PC, SB	Healthy	F: 18	11	7	6	20.4 ± 1.7	21.9 ± 2	23.3 ± 1.2	20.8 ± 1.6	3.6	Control diet
Colakoglu et al. (27)	Turkey	Paralell, R, PC, SB	Healthy	F: 26	12	14	6	21.7 ± 2	20.4 ± 2.5	22.5 ± 1.7	21.6 ± 1.6	3.6	Control diet- exercise
Schmitt et al. (51)	France	Paralell, R, PC, DB	Type 2 diabetes	M/F (F: 10, M: 16)	13	13	12	54.38 ± 8.96	61.62 ± 9.27	32.07 ± 5.37	31.81 ± 4.16	4.5	Control diet
Taylor et al. (52)	United Kingdom	Paralell, R, PC, DB	Healthy	M/F: 40	21	19	12	45 ± 6	47 ± 8	33 ± 3	33 ± 3	4.5	Control diet
Attar-Bashi etval (53)	Australia	Paralell, R, PC	Healthy	M/F: 16	8	8	8	33.1 ± 8.2	37.4 ± 12.2	24 ± 4.3	25 ± 3.8	3.2	Placebo
Lambert et al. (58)	South Africa	Paralell, R, PC, DB	Regularly exercising	F: 37	14	13	12	32 ± 7	32 ± 7	24.2 ± 2.1	24.2 ± 2.1	3.9	Control diet
Nazare et al. (54)	France	Paralell, R, PC, DB	Healthy subjects	M/F: 44	21	23	14	29.4 ± 6.75	28.5 ± 5.7	25.2 ± 1.45	25.1 ± 1.48	3.76	Placebo
Gaullier et al. (55)	Norway	Paralell, R, PC, DB	Overweight and obese	M/F (F: 84, M: 21)	55	50	24	45.8 ± 10	48.7 ± 9.2	30.5 ± 10.4	30.2 ± 10.4	3.4	Placebo
Iwata et al. (59)	japan	Paralell, R, PC, DB	Overweight	M: 40	20	20	12	40.5 ± 8.8	42.5 ± 10.4	28.1 ± 2.1	27.8 ± 1.9	10.8	Placebo
Steck et al. (56)	United Kingdom	Paralell, R, PC, DB	Healthy obese humans	M/F (F: 23, M: 9)	16	16	12	36.3 ± 8.9	34.9 ± 8	32.7 ± 1.8	32.7 ± 1.9	3.2	Placebo
Steck et al. (56)	United Kingdom	Paralell, R, PC, DB	Healthy obese humans	M/F (F: 24, M: 8)	16	16	12	34.1 ± 8.9	34.9 ± 8	32.7 ± 1.7	32.7 ± 1.9	6.4	Placebo
Watras et al. (57)	Canada	Paralell, R, PC, DB	Healthy	M/F (F: 32, M: 8)	22	18	24	34 ± 8	32 ± 7	27.6 ± 1.8	28 ± 2.2	3.2	Placebo
Lambert et al. (58)	South Africa	Paralell, R, PC, DB	Regularly exercising	M: 25	13	12	12	32 ± 7	32 ± 7	22.5 ± 2.5	22.5 ± 2.5	3.9	Control diet
Iwata et al. (59)	japan	Paralell, R, PC, DB	Overweight	M: 40	20	20	12	44.3 ± 10.2	42.5 ± 10.4	27.4 ± 2	27.8 ± 1.9	5.4	Placebo
Park et al. (60)	Korea	Paralell, R, PC, DB	Overweight and obese human	M/F (F: 27, M: 3)	15	15	8	38.7 ± 4.2	40.7 ± 4	25.5 ± 2	26.3 ± 2.5	2.4	Placebo
Aryaeian et al. (61)	Iran	Paralell, R, PC, DB	Rheumatoid arthritis	M/F (F: 38, M: 6)	22	22	12	46.23 ± 13.07	47.95 ± 11.14	27.18 ± 0.99	28.48 ± 0.84	2.5	Placebo
Raff et al. (62)	Denmark	Paralell, R, PC, DB	Healthy young men	M: 38	18	20	5	25.7 ± 4.2	26.1 ± 3.6	22 ± 1.9	22.5 ± 2.1	5.5	Control diet
Kim et al. (63)	Korea	Paralell, R, PC, DB	Healthy overweight women	F: 27	15	12	12	26.33 ± 9.4	29.5 ± 10.8	25.23 ± 2.16	26.47 ± 1.8	3	Control diet
Son et al. (64)	China	Paralell, R, PC, DB	Women with high body fat mass	F: 32	16	16	12	21.9 ± 2.7	21.9 ± 2.7	21.8 ± 1.1	22.5 ± 1.7	4.5	Placebo-exercise
Son et al. (64)	China	Paralell, R, PC, DB	Women with high body fat mass	F: 29	16	13	12	21.9 ± 2.7	21.9 ± 2.7	22.6 ± 1.9	22.8 ± 1.9	4.5	Placebo
Zhao et al. (65)	China	Paralell, R, PC, DB	Obesity-related hypertension	M/F (F: 36, M: 44)	40	40	8	62.3 ± 3.5	59.4 ± 2.4	32.3 ± 2.3	31.2 ± 1.4	4.5	Control diet
Shadman et al. (66)	Iran	Paralell, R, PC, DB	type 2 diabetic patients	M/F (F: 21, M: 18)	19	20	8	45.14 ± 5.77	46.53 ± 4.38	27.4 ± 0.5	27.1 ± 1.8	3	Placebo
Sofi et al. (67)	italy	Crossover, R, PC	Healthy middle-aged	M/F (F: 6, M: 4)	10	10	8	45.6	45.6	25 ± 4	25 ± 4	3	Control diet
Wanders et al. (68)	Netherlands	Crossover, R, PC, SB	Healthy human subjects	M/F (F: 36, M: 25)	61	61	3	30.9 ± 13.7	30.9 ± 13.7	22.8 ± 3.2	22.8 ± 3.2	28.9	Control diet
Michishita et al. (69)	japan	Paralell, R, PC, DB	Healthy Overweight Humans	M/F: 30	15	15	16	34.9 ± 1.4	39.4 ± 3.2	26.1 ± 1.6	25.6 ± 2	1.6	Amino acids
Sluijs et al. (30)	Netherlands	Paralell, R, PC, DB	Overweight and obese adults	M/F (F: 179, M: 167)	173	173	24	58 ± 0.4	58.8 ± 0.5	28 ± 9.45	27.7 ± 12.75	4	Placebo
Venkatramanan et al. (70)	Canada	Crossover, R, PC, SB	Overweight, borderline hyperlipidemic individuals	M/F (F: 5, M: 10)	15	15	8	46.6 ± 2	46.6 ± 2	NR	NR	1.3	Control diet
Brown et al. (71)	USA	Paralrell, R, PC	Health in young women	F: 18	9	9	8	20–40	20–40	19–30	19–30	1.17	Control diet
Sato et al. (72)	japan	Paralell, R, PC, DB	Healthy subjects	M/F (F: 12, M: 12)	12	12	3	22.3 ± 1.5	22.3 ± 1.5	20.2 ± 2	20.2 ± 2	2.2	Control diet
Joseph et al. (73)	Canada	Crossover, R, PC, DB	Overweight, hyperlipidemic	M: 27	27	27	8	18-60	18-60	31.4 ± 4	31.3 ± 4	3.5	Placebo
Joseph et al. (73)	Canada	Crossover, R, PC, DB	Overweight, hyperlipidemic	M: 27	27	27	8	18-60	18-60	31.5 ± 4	31.3 ± 4	3.5	Placebo
Pfeuffer et al. (74)	Germany	Paralell, R, PC, DB	Obese male subjects	M: 40	21	19	4	45–68	45–68	28.3 ± 2.3	27.8 ± 1.3	4.5	Control diet
Rubin et al. (75)	Germany	Crossover, R, PC, DB	Middle-aged men	M: 35	35	35	4	45-68	45-68	26 ± 3.5	26.1 ± 3	4.25	Control diet
Rubin et al. (75)	Germany	Crossover, R, PC, DB	Middle-aged men	M: 35	35	35	4	45-68	45-68	26 ± 2.6	26.1 ± 3	4.25	Control diet
Chen et al. (76)	Taiwan	Paralell, R, PC, DB	Healthy	M/F (F: 42, M: 21)	30	33	12	33.1 ± 1.1	32.5 ± 1.1	27.56 ± 2.45	28.04 ± 2.94	1.7	Placebo
Carvalho et al. (77)	Brazil	Paralell, R, PC, DB	Metabolic syndrome	F: 14	7	7	12	40 ± 14.12	42 ± 5.16	32.53 ± 2.1	32.3 ± 2.16	3	Placebo
Lopez-Plaza et al. (78)	Spain	Paralell, R, PC, DB	Healthy overweight people	M/F (F: 29, M: 9)	22	16	24	43 ± 8.3	44.35 ± 7.79	28.44 ± 1.08	28.56 ± 0.95	3	Placebo
Bulut et al. (79)	Turkey	Paralell, R, PC, DB	Young men	M: 18	9	9	4	19–31	19–31	27.5 ± 2.6	26.8 ± 1.9	3	Placebo
Shadman et al. (80)	Iran	Paralell, R, PC, DB	Overweight type2 diabetics	M/F (F: 21, M: 18)	19	20	8	45.1 ± 5.7	45.5 ± 4.3	27.4 ± 0.5	27.1 ± 1.8	3	Placebo
Jenkins et al. (81)	USA	Paralell, R, PC, DB	Moderately trained men	M: 34	18	16	6	21.5 ± 2.8	21.5 ± 2.8	NR	NR	5.63	Placebo
Eftekhari et al. (31)	Iran	Paralell, R, PC	Atherosclerosis	M/F (F: 31, M:26)	29	28	8	52.79 ± 14.11	55.85 ± 14.13	24.02 ± 2.76	24.66 ± 2.34	3	Control diet
Baghi et al. (82)	Iran	Paralell, R, PC, DB	Athletic	M: 23	13	10	2	18.46 ± 1	18.2 ± 0.5	23.13 ± 0.89	23.83 ± 2.18	5.6	Placebo
Ebrahimi-Mameghani et al. (83)	Iran	Paralell, R, PC, B	Non-alcoholic fatty liver disease	M/F (F:33, M: 5)	19	19	8	36.74 ± 6.87	38.58 ± 8.24	32.72 ± 4.63	35.27 ± 3.46	3	Placebo
Ribeiro et al. (84)	Brasil	Paralell, R, PC, DB	Obese women	F: 28	15	13	8	23.1 ± 2.8	23.2 ± 2.6	28.9 ± 2.6	30.1 ± 3.2	3.2	Placebo
Fouladi et al. (32)	Iran	Paralell, R, PC	Overweight	M/F (F: 62, M: 51)	57	56	12	35 ± 30	35 ± 29	27.6 ± 2.74	27.7 ± 2.98	3	Control diet
Fouladi et al. (32)	Iran	Paralell, R, PC	Overweight	M/F (F: 62, M: 52)	58	56	12	36.5 ± 30	35 ± 29	27.6 ± 2.9	27.7 ± 2.98	3	Control diet
Chang et al. (21)	China	Paralell, R, PC, DB	Healthy adults	M/F (F: 40, M: 25)	32	33	12	25.3 ± 4.3	25.2 ± 4.4	26.4 ± 4.1	26.4 ± 3.2	3.2	Placebo

### Meta-analysis results

#### Effects of CLA supplementation on TG concentration

**Figure 2:**
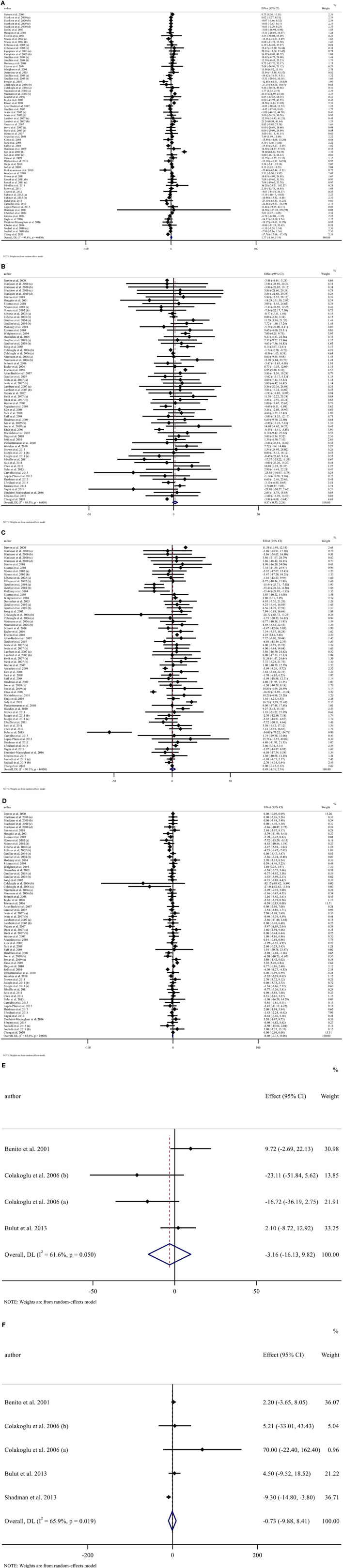
Forest plot presenting mean difference (MD) and 95% confidence intervals for the impact of CLA supplementation on **(A)** TG (mg/dl), **(B)** TC (mg/dl), **(C)** LDL-C (mg/dl), **(D)** HDL-C (mg/dl), **(E)** Apo A (mg/dl), and **(F)** Apo B (mg/dl).

Overall, 73 effect sizes with a total sample size of 3,511 participants (1,773 cases and 1,738 control subjects) were included in the analysis. After combining effect sizes, we found that there was no significant effect of CLA supplementation on TG levels (WMD: 1.76, 95% CI: −1.65, 5.19 mg/dL, *p* = 0.312) (Figure 2A). However, there was a high between-study heterogeneity (*I*^2^: 99.8%, *p* < 0.001). To detect the sources of between-study heterogeneity, we performed subgroup analyses according to baseline levels of TG (≥150 *vs*. <150 mg/dL), length of intervention (≥8 *vs*. <8 weeks), health status of participants (healthy, unhealthy), supplementation dose (≥3 *vs*. <3 g/day), and baseline BMI (normal, overweight, and obese) (Table 3). Subgroup analysis showed that CLA supplementation did not significantly reduce TG concentrations across none of the subgroups.

**Table 3:** Subgroup analyses of CLA supplementation on lipid profile in adults.

	**Number of studies**	**WMD (95%CI)**	***P*-value**	**Heterogeneity**	
				***P* heterogeneity**	***I* ^2^**
**Subgroup analyses of CLA supplementation on TG**					
Overall effect	73	1.76 (−1.65, 5.19)	0.312	< 0.001	99.8%
**Baseline TG (mg/dL)**					
<150	51	1.34 (−4.40, 7.08)	0.648	<0.001	99.8%
≥150	18	3.57 (−8.01, 15.15)	0.546	<0.001	79.0%
**Trial duration (week)**					
≥12	41	3.49 (−0.98, 7.98)	0.126	<0.001	99.9%
<12	32	−0.73 (−6.34, 4.87)	0.798	<0.001	87.4%
**Health status**					
Healthy	60	1.84 (−1.84, 5.53)	0.328	<0.001	99.8%
Unhealthy	13	1.64 (−8.87, 12.15)	0.759	<0.001	82.9%
**Supplementation dose (g/day)**					
≥3	44	2.69 (−1.62, 7.00)	0.221	<0.001	99.7%
<3	29	0.16 (−5.44, 5.76)	0.955	<0.001	99.7%
**Baselin BMI (kg/m** ^**2**^ **)**					
Normal (18.5–24.9)	18	1.85 (−7.82, 11.52)	0.707	<0.001	79.2%
Overweight (25–29.9)	36	2.71 (−2.42, 7.84)	0.301	<0.001	99.9%
Obese (>30)	17	0.05 (−7.02, 7.13)	0.988	<0.001	86.9%
**Subgroup analyses of CLA supplementation on TC**					
Overall effect	67	0.86 (−0.53, 2.26)	0.225	<0.001	89.5%
**Baseline TC (mg/dL)**					
≥200	27	2.22 (−0.36, 4.81)	0.093	<0.001	77.9%
<200	36	0.11 (−1.95, 2.17)	0.914	<0.001	93.1%
**Trial duration (week)**					
≥12	37	1.06 (−0.44, 2.58)	0.168	<0.001	78.9%
<12	30	−0.24 (−2.68, 2.18)	0.841	<0.001	86.3%
**Health status**					
Healthy	54	2.25 (0.61, 3.88)	**0.007**	<0.001	91.1%
Unhealthy	13	−3.26 (−5.88, −0.65)	**0.014**	0.003	60.2%
**Supplementation dose (g/day)**					
≥3	41	−0.10 (−1.43, 1.21)	0.872	<0.001	84.7%
<3	26	1.54 (−1.64, 4.74)	0.341	<0.001	71.8%
**Baselin BMI (kg/m** ^**2**^ **)**					
Normal (18.5–24.9)	18	1.69 (−0.52, 3.92)	0.931	0.003	55.1%
Overweight (25–29.9)	30	0.08 (−1.84, 2.01)	0.135	<0.001	92.6%
Obese (>30)	17	0.12 (−3.46, 3.72)	0.944	<0.001	77.4%
**Subgroup analyses of CLA supplementation on LDL-C**					
Overall effect	66	0.49 (−1.75, 2.74)	0.668	<0.001	96.3%
**Baseline LDL (mg/dL)**					
≥100	49	0.92 (−2.30, 4.14)	0.576	<0.001	94.1%
<100	13	0.07 (−0.75, 0.90)	0.858	0.389	5.8%
**Trial duration (week)**					
≥12	38	1.55 (−1.44, 4.55)	0.310	<0.001	97.5%
<12	28	−1.32 (−5.64, 2.99)	0.547	<0.001	90.3%
**Health status**					
Healthy	53	1.61 (−0.93, 4.16)	0.214	<0.001	96.6%
Unhealthy	12	−3.59 (−9.00, 1.82)	0.193	<0.001	90.2%
**Supplementation dose (g/day)**					
≥3	39	0.32 (−2.80, 3.46)	0.838	<0.001	97.6%
<3	27	0.91 (−2.25, 4.09)	0.571	<0.001	83.1%
**Baselin BMI (kg/m** ^**2**^ **)**					
Normal (18.5–24.9)	17	3.39 (1.56, 5.22)	**<0.001**	0.586	0.0%
Overweight (25–29.9)	31	0.14 (−3.09, 3.38)	0.929	<0.001	98.1%
Obese (>30)	17	−0.15 (−5.71, 5.40)	0.956	<0.001	87.3%
**Subgroup analyses of CLA supplementation on HDL-C**					
Overall effect	67	−0.40 (−0.72, −0.07)	**0.015**	<0.001	63.0%
**Baseline HDL (mg/dL)**					
≥50	39	−0.19 (−0.60, 0.22)	0.361	<0.001	69.4%
<50	24	−0.66 (−1.37, 0.03)	0.063	0.007	46.2%
**Trial duration (week)**					
≥12	38	−0.06 (−0.30, 0.17)	0.586	0.010	38.2%
<12	29	−0.81 (−2.01, 0.39)	0.186	0.007	73.9%
**Health status**					
Healthy	54	−0.24 (−0.55, 0.05)	0.108	<0.001	50.2%
Unhealthy	13	−0.66 (−2.23, 0.89)	0.403	<0.001	82.2%
**Supplementation dose (g/day)**					
≥3	40	−0.07 (−0.41, 0.27)	0.691	<0.001	59.2%
<3	27	−0.98 (−1.95, −0.01)	**0.048**	<0.001	62.5%
**Baselin BMI (kg/m** ^**2**^ **)**					
Normal (18.5–24.9)	18	−1.68 (−3.17, −0.19)	**0.026**	0.114	29.7%
Overweight (25–29.9)	31	−0.19 (−0.47, 0.08)	0.175	<0.001	56.1%
Obese (>30)	17	−0.58 (−2.17, 1.01)	0.476	<0.001	76.2%
**Subgroup analyses of CLA supplementation on Apo A**					
	4	−3.15 (−16.12, 9.81)	0.634	0.050	61.6%
**Subgroup analyses of CLA supplementation on Apo B**					
	5	−0.73 (−9.87, 8.41)	0.875	0.019	65.9%

CI, confidence interval; WMD, weighted mean differences. *Bold value: significant effect (*P* < 0.05).

#### Effects of CLA supplementation on TC concentration

Sixty-seven arms of RCTs (1,561 cases and 1,529 control subjects) reported the effects of CLA supplementation on TC levels, and combining effect sizes from these studies showed a non-significant effect of CLA intake on TC concentrations (WMD: 0.86, 95% CI: −0.53, 2.26 mg/dL, *p* = 0.225), with a considerable between-study heterogeneity (*I*^2^: 89.5%, *p* < 0.001) (Figure 2B). To find the probable source of heterogeneity, subgroup analysis was applied. All of the abovementioned subgroup analysis indicated that health status subgroups could explain study heterogeneity (Table 3).

#### Effects of CLA supplementation on LDL-C concentration

Considering 66 effect sizes that included 3,217 participants (1,627 cases and 1,590 control subjects), no significant effect of CLA supplementation on serum concentrations of LDL-C was found (WMD: 0.49, 95% CI: −1.75, 2.74 mg/dL, *p* = 0.668). However, there was a considerable between-study heterogeneity (*I*^2^: 96.3%, *p* < 0.001) (Figure 2C). Subgroup analysis also revealed that CLA supplementation significantly increased serum LDL-C level across the individuals with normal BMI (*p* < 0.001) (Table 3).

#### Effects of CLA supplementation on HDL-C concentration

Totally, 67 effect sizes with a sample size of 3,283 participants (1,658 cases and 1,625 control subjects) were included in the analysis. Combining these effect sizes, a significant reduction was seen in serum concentrations of HDL-C following CLA supplementation (WMD: −0.40, 95% CI: −0.72, −0.07 mg/dL, *p* = 0.015) (Figure 2D). There was evidence of moderate between-study heterogeneity (*I*^2^: 63%, *p* < 0.001). In the subgroup analysis, we found that the effect of CLA supplementation on serum HDL-C concentrations strengthened in studies performed on individuals with normal BMI and when the supplementation dose of CLA is <3 g/day (Table 3).

#### Effects of CLA supplementation on Apo A

Four effect sizes including 79 participants (39 cases and 40 control subjects) provided information on Apo A as an outcome measure. Pooled results showed that CLA intake did not significantly affect Apo A (WMD: −3.15, 95% CI: −16.12, 9.81, *p* = 0.634) (Figure 2E), with a moderate heterogeneity among studies (*I*^2^: 61.6%, *p* = 0.050) (Table 3).

#### Effects of CLA supplementation on Apo B

Five effect sizes including a total of 118 participants (58 cases and 60 control subjects) indicated the effects of CLA intake on Apo B. CLA supplementation did not significantly decrease Apo B (WMD: −0.73, 95% CI: −9.87, 8.41, *p* = 0.875), without significant heterogeneity between studies (*I*^2^: 65.9%, *p* = 0.019) (Figure 2F and Table 3).

### Publication bias

**Figure 3:**
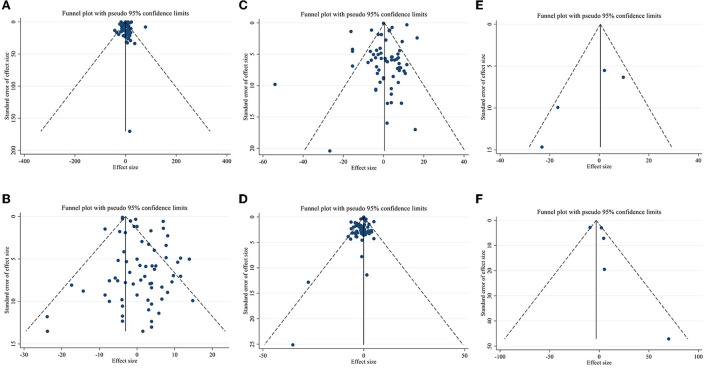
Funnel plots for **(A)** TG (mg/dl), **(B)** TC (mg/dl), **(C)** LDL-C (mg/dl), **(D)** HDL-C (mg/dl), **(E)** Apo A (mg/dl), and **(F)** Apo B (mg/dl).

Publication bias assessment was performed based on visual inspection of funnel plot, Begg's and Egger's linear regression test. Results revealed no publication bias for TG (*p* = 0.646), LDL-C (*p* = 0.578), Apo A (*p* = 0.148), and Apo B (*p* = 0.340) based on Egger's test. However, there was publication bias for TC (*p* = 0.001, Begg's test) (*p* = 0.002, Egger's test). Furthermore, there was no evidence of a substantial publication bias for HDL-C (*p* = 0.858) based on Begg's test (Figure 3).

### Linear and non-linear dose-responses between dose and duration of CLA supplementation and lipid profile components

**Figure 4:**
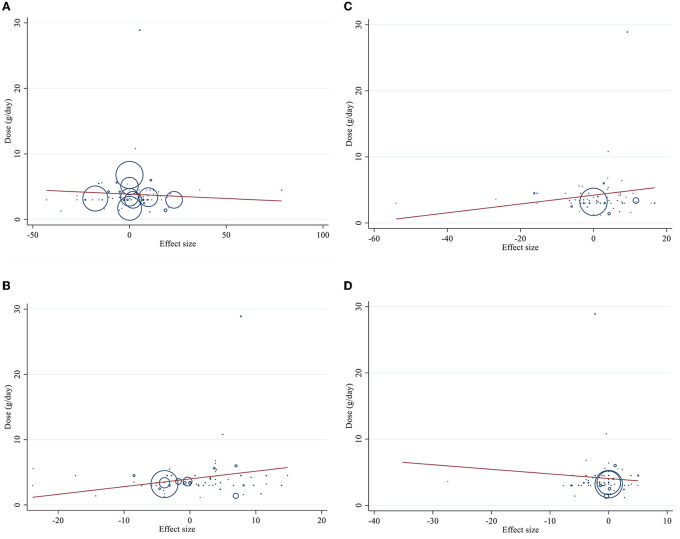
Linear meta regression plots based on dose (g/d) of intervention for **(A)** TG (mg/dl), **(B)** TC (mg/dl), **(C)** LDL-C (mg/dl), and **(D)** HDL-C (mg/dl).

**Figure 5:**
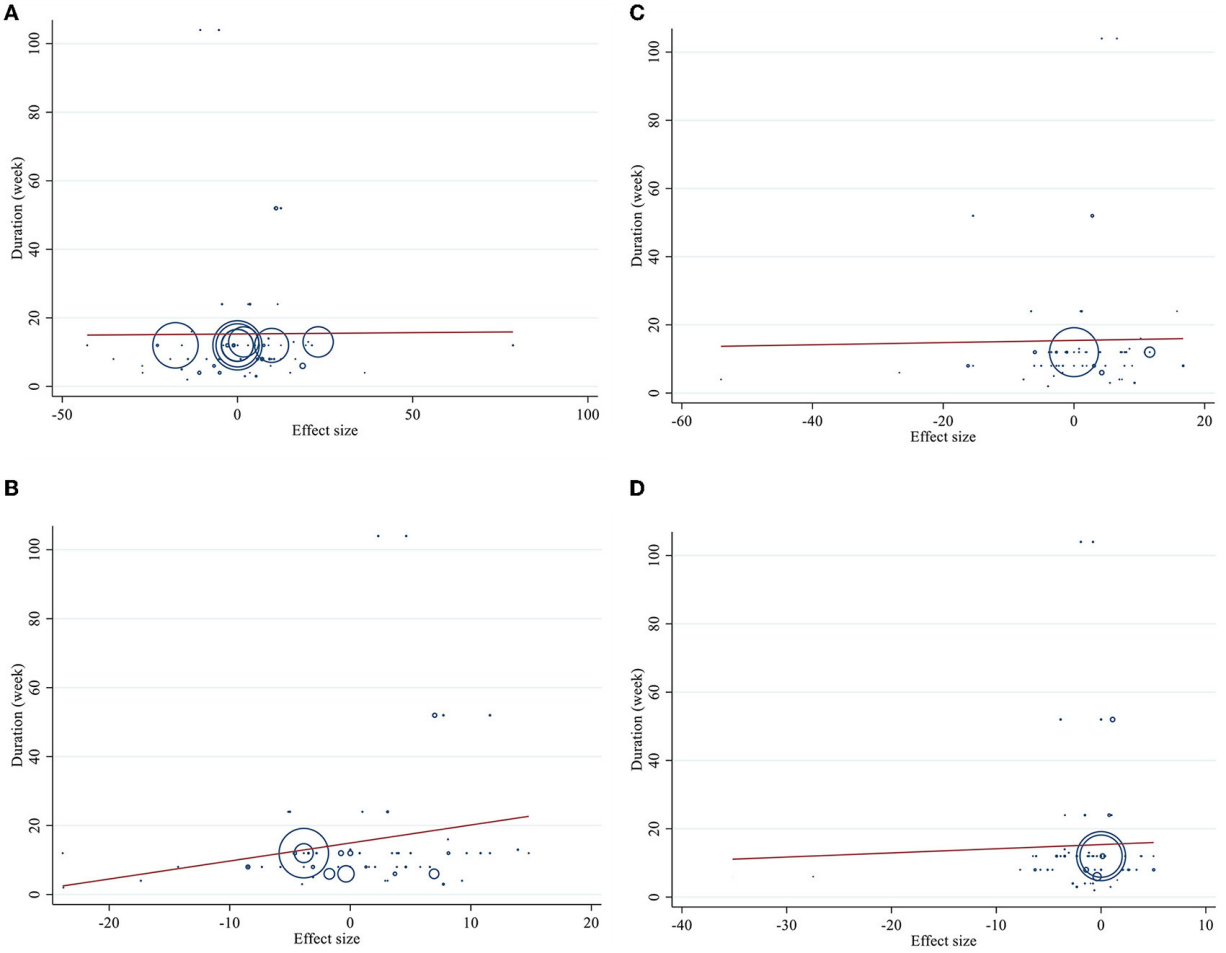
Linear meta regression plots based on duration (week) of intervention for **(A)** TG (mg/dl), **(B)** TC (mg/dl), **(C)** LDL-C (mg/dl), and **(D)** HDL-C (mg/dl).

Meta-regression using the random-effects model was undertaken to investigate the potential association between a change in lipid profile and dose of CLA (g/day) and duration of intervention. Meta-regression analysis indicated that there was not a linear association between absolute changes in all the factors and duration and dose (Figures 4, 5).

**Figure 6:**
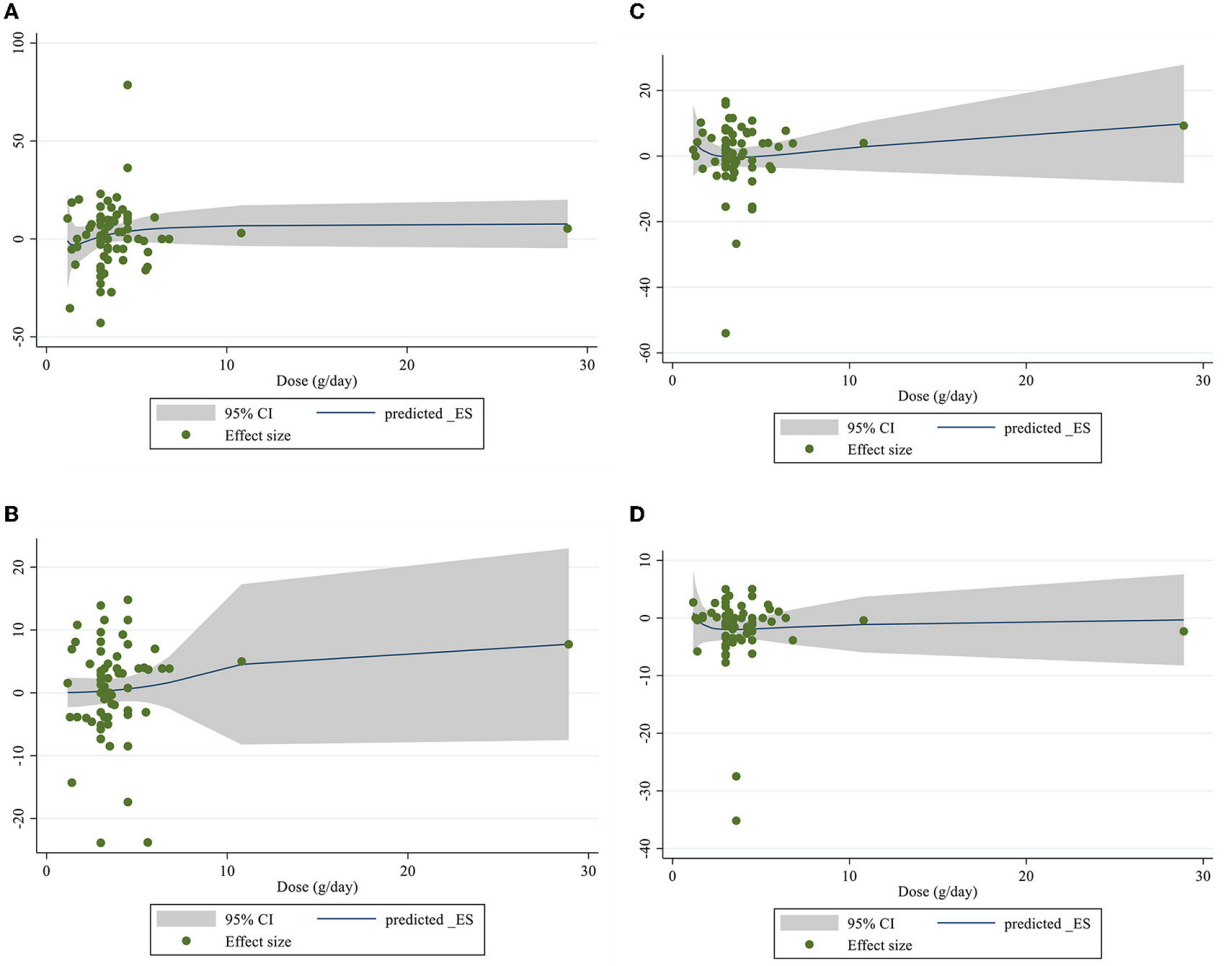
Non-linear dose-respons plots based on dose (g/d) of intervention for **(A)** TG (mg/dl), **(B)** TC (mg/dl), **(C)** LDL-C (mg/dl), and **(D)** HDL-C (mg/dl).

**Figure 7:**
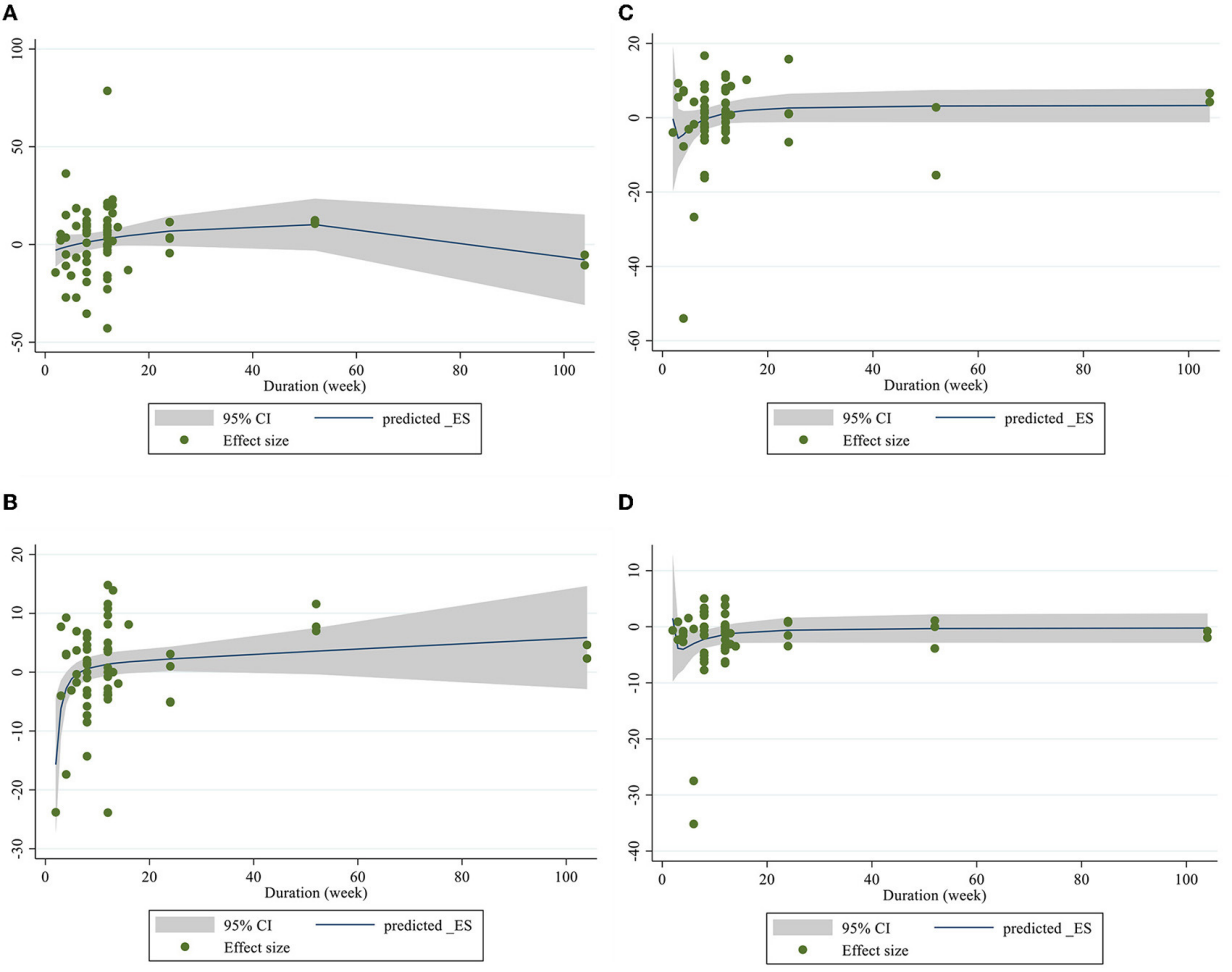
Non-linear dose-respons plots based on duration (week) of intervention for **(A)** TG (mg/dl), **(B)** TC (mg/dl), **(C)** LDL-C (mg/dl), and **(D)** HDL-C (mg/dl).

Dose–response analysis showed that CLA supplementation changed TC significantly based on duration (*r* = −0.006, P-non-linearity = 0.009) in non-linear fashion. Additionally, significant associations were not observed for other outcomes in non-linear dose–responses (Figures 6, 7).

### Grading of evidence

The GRADE protocol was applied for the assessment of the certainty of the evidence (Table 4) and determined the evidence regarding HDL-C to be of moderate quality, owing to serious inconsistency and TG, LDL-C, Apo A, and Apo B to be of low quality for a serious imprecision and inconsistency reason. However, the evidence relating to TC was downgraded to very low quality, because of the serious inconsistency, imprecision, and publication bias.

**Table 4:** GRADE profile of CLA supplementation for on lipid profile.

**Outcomes**	**Risk of bias**	**Inconsistency**	**Indirectness**	**Imprecision**	**Publication bias**	**Number of intervention/** **control**	**Quality of evidence**
TG	No serious limitation	Serious limitation^a^	No serious limitation	Serious limitation^b^	No serious limitation	3,511 (1,773/1,738)	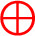 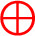 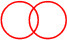 Low
TC	No serious limitation	Serious limitation^a^	No serious limitation	Serious limitation^b^	Serious limitation^c^	3,090 (1,561/1,529)	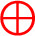 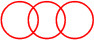 Very Low
LDL-C	No serious limitation	Serious limitation^a^	No serious limitation	Serious limitation^b^	No serious limitation	3,217 (1,627/1,590)	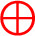 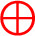 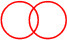 Low
HDL-C	No serious limitation	Serious limitation^a^	No serious limitation	No serious limitation	No serious limitation	3,283 (1,658/1,625)	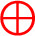 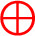 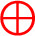 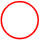 Moderate
Apo A	No serious limitation	Serious limitation^a^	No serious limitation	Serious limitation^b^	No serious limitation	79 (39/40)	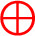 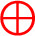 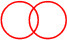 Low
Apo B	No serious limitation	Serious limitation^a^	No serious limitation	Serious limitation^b^	No serious limitation	118 (58/60)	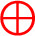 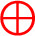 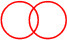 Low

a There is significant heterogeneity for TG (I^2^ = 99.8%), TC (I^2^ = 89.5%), LDL-C (I^2^ = 96.3%), HDL-C (I^2^ = 63.0%), Apo A (I^2^ = 61.6%), and Apo B (I^2^ = 65.9%).

b There is no evidence of significant effects of CLA supplementation on TG, TC, LDL-C, Apo A, and Apo B.

c There is significant publication bias for TC (p = 0.001).

### Sensitivity analysis

The sensitivity analysis was also carried out to examine the impact of each individual study on the pooled effect size by removing each study in turn. The sensitivity analysis showed that the result was not significantly influenced by any of the studies assessing the TG, TC, and LDL-C levels. However, the effect of CLA on HDL-C was significantly changed after removing studies by Risérus et al. (41) (WMD: −0.31, 95% CI: −0.63, 0.00) (41), Sofi et al. (67) (WMD: −0.22, 95% CI: −0.52, 0.06) (67), and Eftekhari et al. (31) (WMD: −0.30, 95% CI: −0.63, 0.02) (31).

## Discussion

In this systematic review and meta-analysis, we aimed to evaluate the effects of CLA supplementation on lipid profile. The results derived from this study suggest that the administration of CLA has non-significant effects on serum levels of TG, TC, LDL, Apo A, and Apo B. However, CLA supplementation decreases HDL statistically but not clinically.

Initial animal studies suggested that CLA promotes significant changes to lipid metabolism *in vivo*. In mice, it has been reported that CLA decreases cholesterol levels (90–93) and increases HDL levels (92, 94, 95), suggesting that CLA could impact cholesterol efflux. It should be noted that mice inherently have a much different lipoprotein profile than humans, where the majority of the cholesterol is carried on HDL rather than both LDL and HDL (96). However, the proven effects of CLA administration on lipid profile are unclear, as clinical studies on this topic have produced inconsistent results. A meta-analysis of 23 studies by Derakhshande-Rishehri et al. showed that CLA supplementation has favorable effects on LDL cholesterol levels without any changes in TC, TG, and HDL (28). They also reported that consumption of foods enriched with CLA has similar effects. In a more recent meta-analysis of 13 studies by Moreno et al. they showed that CLA was associated with a reduction in HDL-C levels and an increase in triglyceride levels (97). However, our results of 56 studies CLA supplementation not only cannot improve LDL but also it can decrease HDL, in inconsistence with their findings. Differences in the different number of studies, the use of different doses and type of supplementation can be the explanations for inconsistencies between our findings and Derakhshande-Rishehri et al. findings.

HDL-lowering effects of CLA are in contrast with the previously mentioned findings from mechanistic studies. Therefore, the possible mechanisms underlying the negative effects of CLA supplementation on HDL concentrations remain unclear. Because low HDL levels are an independent risk factor for cardiovascular events (98), the current reduction of 0.4 mg/dl with CLA is of clinical concern. Riserus et al. hypnotized that HDL-lowering effect of CLA may be related to its leptin-lowering effect (41). According to their findings, the decrease in HDL cholesterol following CLA supplementation was correlated with a change in leptin. Because of the importance of HDL-decrement which is reported in our study as well as some previous studies, further mechanistic studies are needed to the possible mechanism underlying the effects of CLA supplementation on lipid profile.

The present meta-analysis contains some strengths and limitations. The main strength of this study is the relatively acceptable number of studies (*N* = 56) and high sample size compared with previous meta-analyses (*N* = 13 and 23). Moreover, we analyzed a wider range of lipid profile biomarkers (TC, TG, LDL, HDL, and apo A and apo B). Another advantage is the lack of publication bias in almost all analyses (all except for TC). Furthermore, we performed a dose–response analysis to evaluate the association between pooled effect size, dosage, and duration of CLA supplementation.

Another strength of this study relates to the inclusion of several long-term studies, which certainly has the advantage of documenting the long-term effects of CLA administration on lipid profile and allowing comparisons to shorter duration designs. Finally, we graded the overall certainty of evidence across the studies according to the GRADE guidelines. Regarding limitations, statistical heterogeneity is apparent in our analysis. This may be attributed to methodological diversity (different study designs) and/or differences in treatment regimens (doses/durations) or the intervention type. In addition, the quality of evidence regarding all markers was identified as very low to moderate quality.

Overall, the results of the current systematic review and meta-analysis demonstrate that supplementation of CLA statistically decreases HDL but not clinically. However, CLA may not affect serum levels of TG, TC, LDL, apo-A, and apo-B. However, given the low quality of some of the included studies, further studies are needed to support the veracity of our findings.

## Data availability statement

The original contributions presented in the study are included in the article/supplementary material, further inquiries can be directed to the corresponding author/s.

## Author contributions

OA contributed in conception and design of the study, data analysis, and supervised the study. DA-l and KN contributed to data extraction. MZ contribute to correct the proof of manuscript. SS and MR screened articles for inclusion criteria. SD and NH contributed in manuscript drafting. MN contributed to edit English language. All authors approved the final version of the manuscript.

## Funding

This work was supported by Shiraz University of Medical Sciences (grant number: 26331).

## Conflict of interest

The authors declare that the research was conducted in the absence of any commercial or financial relationships that could be construed as a potential conflict of interest.

## Publisher's note

All claims expressed in this article are solely those of the authors and do not necessarily represent those of their affiliated organizations, or those of the publisher, the editors and the reviewers. Any product that may be evaluated in this article, or claim that may be made by its manufacturer, is not guaranteed or endorsed by the publisher.
